# Supplementary figures and images for: Isolation and in silico analysis of a new subclass of parasporin 4 from Bacillus thuringiensis coreanensis
Source: PeerJ. 2025 Mar 24;13:e19061. doi: 10.7717/peerj.19061 (PMC11949118; doi:10.7717/peerj.19061)

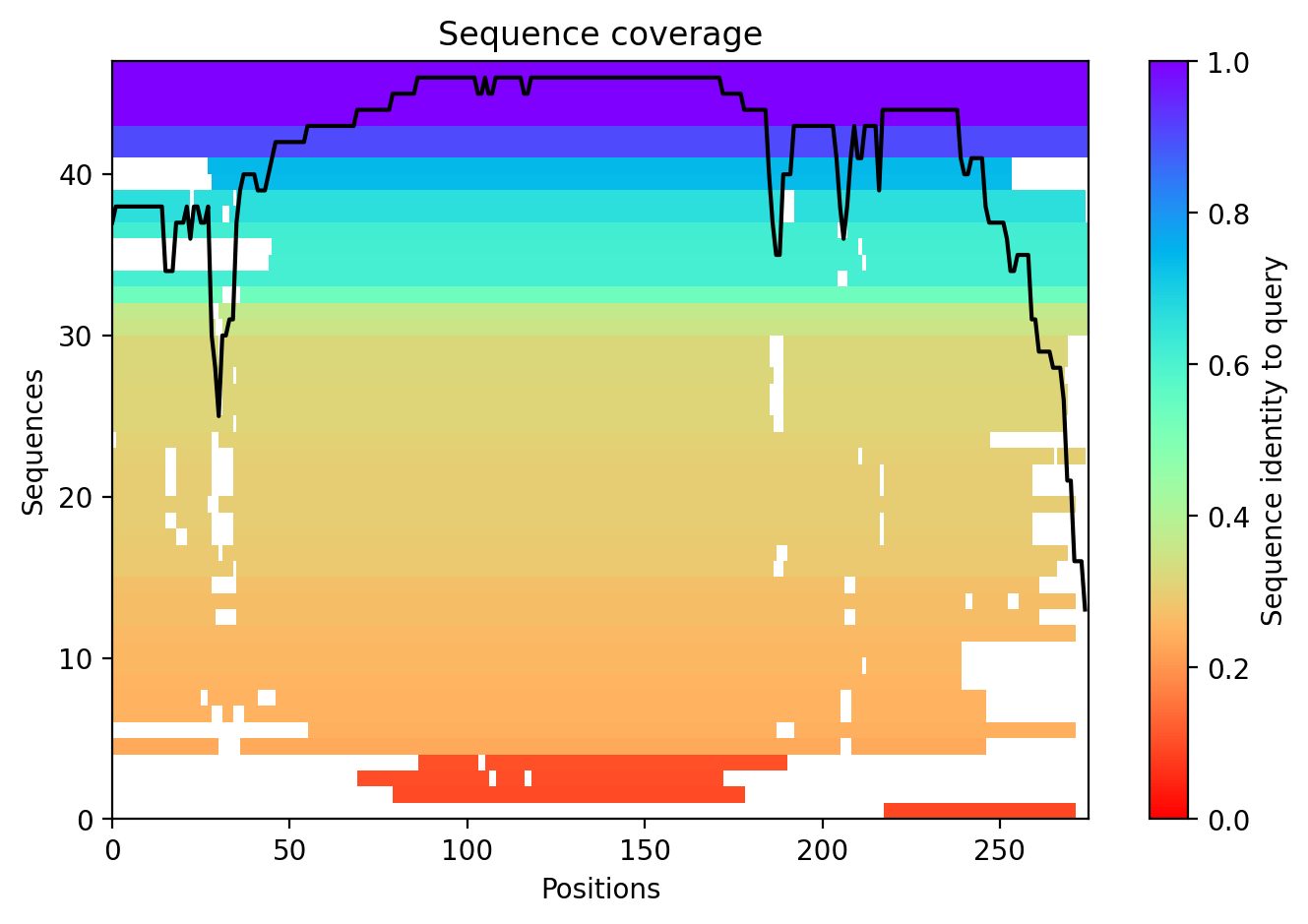

Supplement: Supplemental Information 5 — These data were obtained from modeling the PS4Ab1 protein using the Alphafold2 tool, its files can be opened in Pymol, Notepad and common image viewer. [file peerj-13-19061-s005.zip › PS4Ab1 protein modeling file/bscillud_6c2f1_coverage.png]

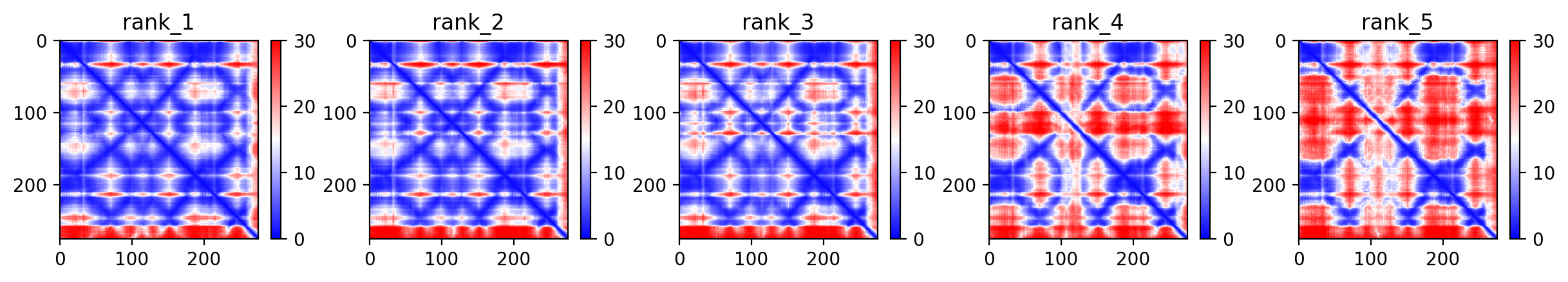

Supplement: Supplemental Information 5 — These data were obtained from modeling the PS4Ab1 protein using the Alphafold2 tool, its files can be opened in Pymol, Notepad and common image viewer. [file peerj-13-19061-s005.zip › PS4Ab1 protein modeling file/bscillud_6c2f1_pae.png]

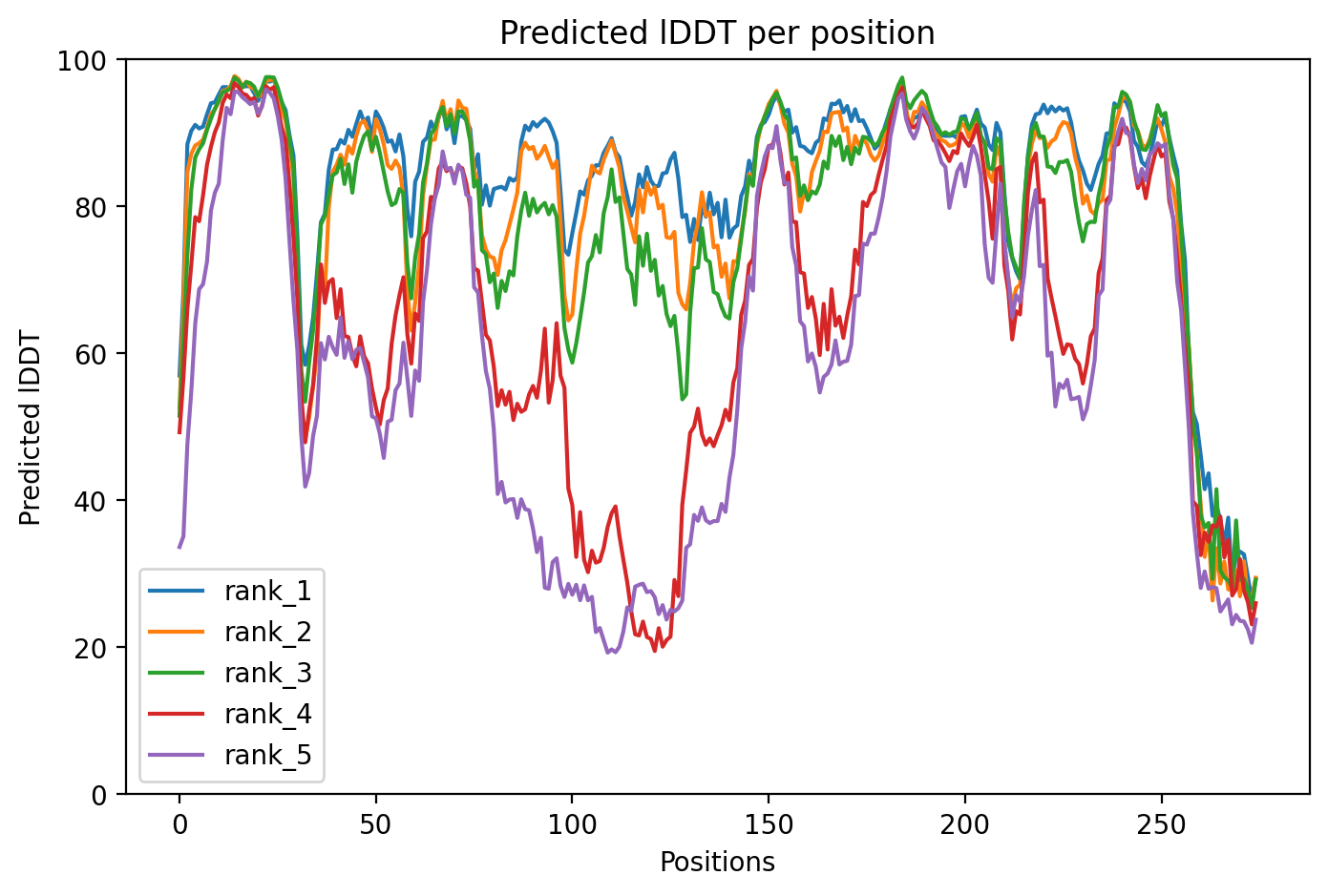

Supplement: Supplemental Information 5 — These data were obtained from modeling the PS4Ab1 protein using the Alphafold2 tool, its files can be opened in Pymol, Notepad and common image viewer. [file peerj-13-19061-s005.zip › PS4Ab1 protein modeling file/bscillud_6c2f1_plddt.png]
