# Supplementary material for: Isolation and in silico analysis of a new subclass of parasporin 4 from Bacillus thuringiensis coreanensis
Source: PeerJ. 2025 Mar 24;13:e19061. doi: 10.7717/peerj.19061 (PMC11949118; doi:10.7717/peerj.19061)
Supplement: Supplemental Information 7 [file peerj-13-19061-s007.zip › Ramachandran for all models/Modelo 5.pdf]

PROCHECK

Ramachandran Plot

saves

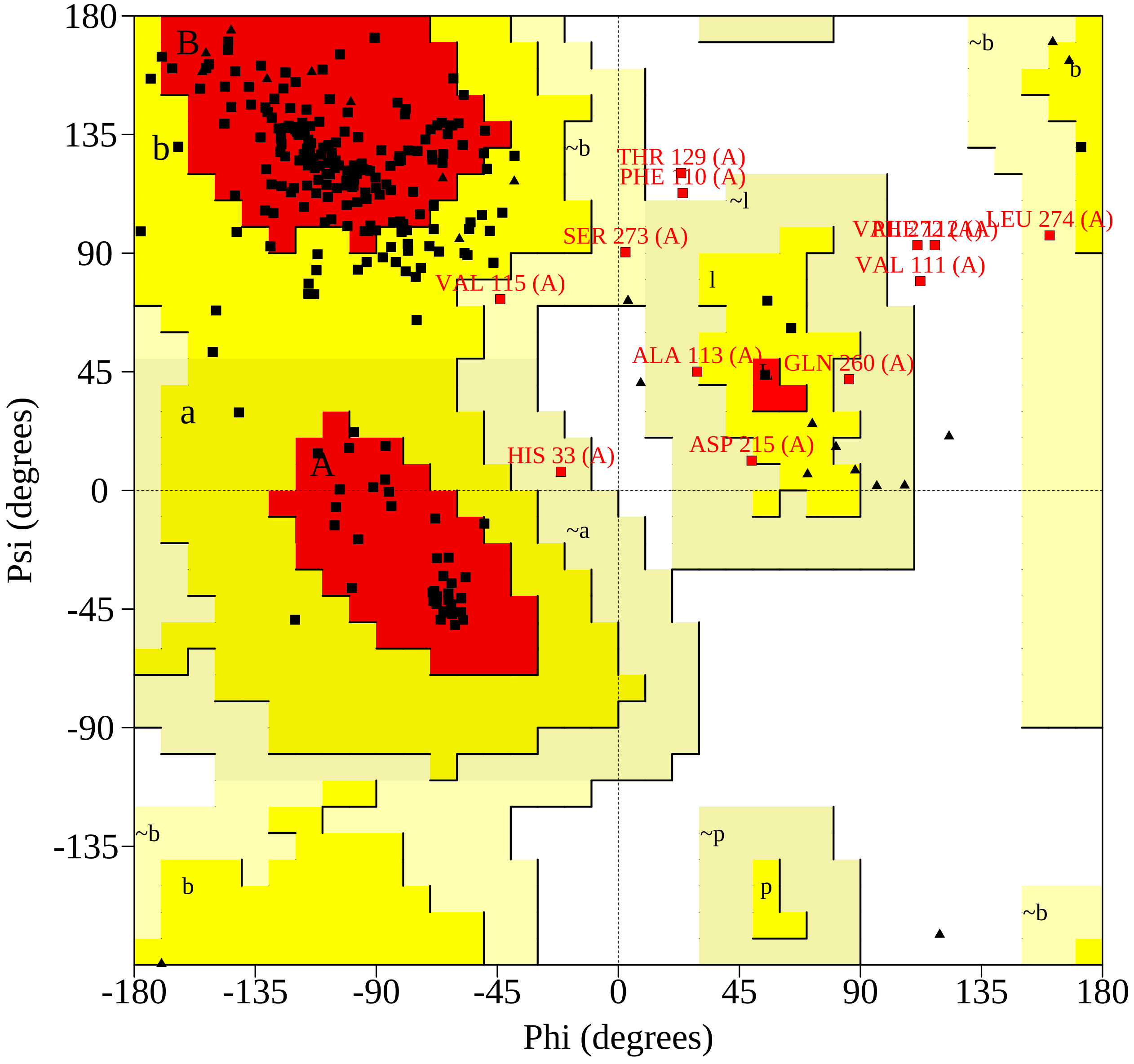

Plot statistics

Residues in most favoured regions [A,B,L]	178	76.4%
Residues in additional allowed regions [a,b,l,p]	43	18.5%
Residues in generously allowed regions [~a,~b,~l,~p]	7	3.0%
Residues in disallowed regions	5	2.1%
	----	------
Number of non-glycine and non-proline residues	233	100.0%
Number of end-residues (excl. Gly and Pro)	2	
Number of glycine residues (shown as triangles)	24	
Number of proline residues	16	

Total number of residues	275	

Based on an analysis of 118 structures of resolution of at least 2.0 Angstroms and R-factor no greater than 20%, a good quality model would be expected to have over 90% in the most favoured regions.

saves_01.ps
